# When aging switches on Alzheimer’s

**DOI:** 10.18632/aging.203085

**Published:** 2021-05-20

**Authors:** Yue Dong, Benjamin A. Harlan, Gregory J. Brewer

**Affiliations:** 1 Department of Biomedical Engineering, MIND Institute, Center for Neurobiology of Learning and Memory, University of California, Irvine, CA 92697, USA; 2 Department of Cell and Molecular Pharmacology and Experimental Therapeutics, Medical University of South Carolina, Charleston, SC 29425, USA

**Keywords:** aging, Alzheimer’s disease, NAD+/NADH, oxidative shift, mitochondrial impairment, neuroinflammation

Aging increases the risk for developing Alzheimer’s disease (AD). Pathological hallmarks of AD include abnormal deposits of extracellular beta amyloid (Aβ) plaques and intracellular neurofibrillary tangles, which are proposed to impair synaptic function to foster progressive cognitive impairment. By 2050, 7 million people above age 85 in U.S. are projected to have Alzheimer’s dementia, accounting for 51% of the population older than 65 [1]. Although Aging and AD undeniably share a number of common features, such as oxidative stress, mitochondrial impairment, bioenergetic and metabolic shifts, AD is not the inevitable co-morbidity of aging. This escape from AD arouses hope that anti-aging interventions could decelerate aging switches for AD dementia.

The epigenetic oxidative redox shift (EORS) theory of aging proposed a sedentary behavior in old age that triggers an oxidative shift and mitochondrial impairment (Figure 1) [2]. Epigenetic marks get set for low mitochondrial capacity and minimal energy production. To maintain resting redox energy levels, aerobic glycolysis and lactate production are upregulated. Epigenetic enforcement of metabolic shifts reinforces a sedentary behavior to form a vicious cycle. Mitochondrial function and NAD homeostasis decline with age in brains of healthy volunteers. Several interventions can decelerate this cycle, including exercise, redox agents, epigenetic modulators and senolytic drugs. The reduced form of nicotinamide adenine dinucleotide (NADH) serves as the intracellular redox-energy currency because of its role as the substrate for ATP generation via oxidative phosphorylation (OXPHOS) in mitochondria. The depleted levels of NADH in mitochondria, together with an oxidative shift in NAD^+^/NADH redox state in aging and AD, initiate epigenetic controls for downstream metabolic shifts to compensate for bioenergetic deficits and redox imbalance [3,4]. Supplementation of NAD precursors, such as nicotinamide, nicotinamide riboside or nicotinamide mononucleotide as redox-energy modulators, ameliorates the bioenergetic deficits, restores stem cell replication, reverses cognitive decline and extends lifespan in model organisms.

**Figure 1:**
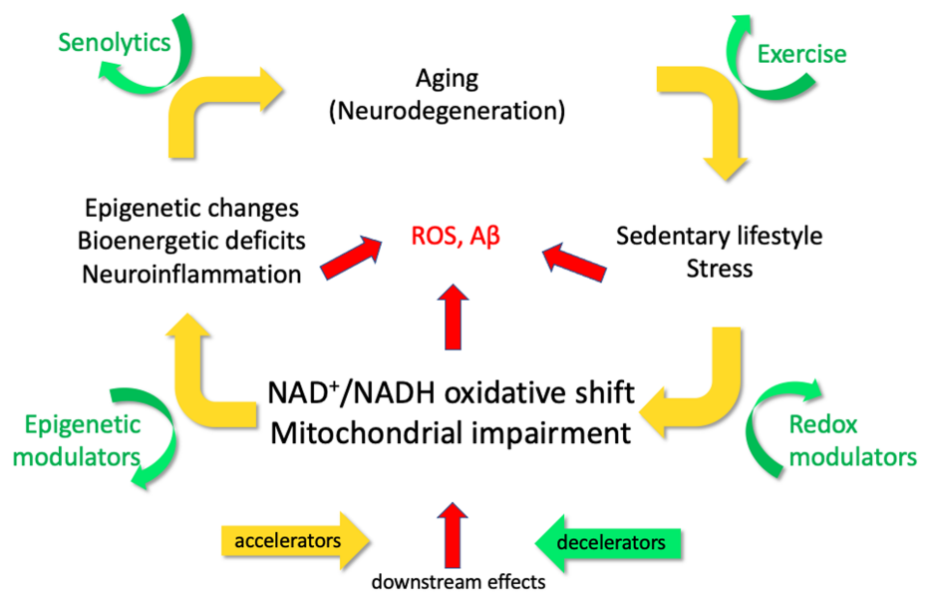
**The EORS downward spiral of aging and Alzheimer’s (Epigenetic Oxidative Redox Shift) [****2****].** Yellow arrows illustrate mechanistic targets that propel the vicious cycle triggered by age-related oxidative shifts (NAD+/NADH) and age-related sedentary behavior, which elicit ROS, inflammatory stress and possibly Aβ. Green arrows indicate practical interventions to decelerate the vicious cycle and impede the progression of aging and AD.

Our environment, lifestyle, stress, physical activity, and habits all modulate epigenetic control of gene expression for continuous environmental tracking. Age-related redox stress, often measured as oxidative stress in aging and AD launches a global switch in the epigenetic landscape, widely affecting methylation, histone modification, and noncoding RNA regulation [5], to further drive downstream metabolic and energetic shifts. These “switches” highlight the potential of epigenetic targets for AD treatment. For instance, Nativio et al. [5] found the histone acetylation mark H4K16ac enriched in the lateral temporal lobe of normal aging, while significantly lost in AD cases. An oxidative shift alters activities of numerous redox-sensitive transcription factors, enzymes and signaling proteins with redox-sensitive cysteines. Global metabolic profiling in the 3xTg-AD mouse model compared to non-transgenic mice found an intense bioenergetic shortage in aging and AD brains [6] with remarkable upregulation of energetic substrates, fatty acids and β-oxidation. Redox-energy deficits direct compensatory shifts in energy-producing pathways of glycolysis and TCA cycle sensed at NAD^+^/NADH redox sites to maintain ATP generation [6].

According to a modified amyloid cascade hypothesis, amyloid-mediated oxidative stress triggers a cascade of downstream effects including mitochondrial dysfunction, excitotoxicity, synaptic loss and neuroinflammation [7]. However, the failure of anti-amyloid and anti-inflammatory therapy in clinical trials allows us to entertain other causal possibilities including an age-related oxidative redox shift as an upstream switch that changes amyloid processing, deposition or clearance. Intriguingly, some resilient older individuals present with similar loads of Aβ and tangles compared to AD cases without experiencing dementia. Further studies in resilient brains point out distinct upregulation of anti-inflammatory cytokines in entorhinal cortex, increased expression of neurotrophic factors and reduced expression of chemokines linked to microglial recruitment, which all suggest activated neuro-glial inflammation in non-resilient AD [8]. Since inflammation is switched on by an oxidative redox state, normal microglia that selectively remove excitotoxic synapses could be over-activated toward inflammatory neurodegeneration in AD. Suitable redox markers could enable measured redox therapies to decelerate inflammation and the neurodegenerative cascade.

Yet the complex mechanisms of switching on so many AD pathologies remain underexplored. While studies on these “switches” enable elucidation of the underlying mechanisms for when aging switches on Alzheimer’s degeneration, more importantly, these “switches” of redox, epigenetics and neuroinflammation encourage early interventions to decelerate AD pathology and retain functional memory.

## References

[1] Alzheimer’s Association. Alzheimers Dement. 2020. 10.1002/alz.12068

[2] Brewer GJ. Exp Gerontol. 2010; 45:173–79. 10.1016/j.exger.2009.11.007 19945522 PMC2826600

[3] Dong Y, et al. Geroscience. 2019; 41:51–67. 10.1007/s11357-019-00052-8 30729413 PMC6423217

[4] Dong Y, et al. Sci Rep. 2019; 9:11274. 10.1038/s41598-019-47582-x 31375701 PMC6677822

[5] Nativio R, et al. Nat Genet. 2020; 52:1024–35. 10.1038/s41588-020-0696-0 32989324 PMC8098004

[6] Dong Y, Brewer GJ. J Alzheimers Dis. 2019; 71:119–40. 10.3233/JAD-190408 31356210 PMC6839468

[7] Butterfield DA, et al. J Neurochem. 2019; 151:459–87. 10.1111/jnc.14589 30216447 PMC6417976

[8] Barroeta-Espar I, et al. Neurobiol Dis. 2019; 121:327–37. 10.1016/j.nbd.2018.10.009 30336198 PMC6437670

